# Glycosylation and raft endocytosis in cancer

**DOI:** 10.1007/s10555-020-09880-z

**Published:** 2020-05-09

**Authors:** Ludger Johannes, Anne Billet

**Affiliations:** 1 Cellular and Chemical Biology Unit, INSERM U1143, CNRS UMR3666, Institut Curie, PSL Research University, 26 rue d’Ulm, 75248 Paris Cedex 05, France; 2 Université de Paris, F-75005 Paris, France

**Keywords:** Glycosphingolipid, GPI-anchored protein, Actin, Cholesterol, Shiga toxin, Cholera toxin

## Abstract

Changes in glycosylation on proteins or lipids are one of the hallmarks of tumorigenesis. In many cases, it is still not understood how glycan information is translated into biological function. In this review, we discuss at the example of specific cancer-related glycoproteins how their endocytic uptake into eukaryotic cells is tuned by carbohydrate modifications. For this, we not only focus on overall uptake rates, but also illustrate how different uptake processes—dependent or not on the conventional clathrin machinery—are used under given glycosylation conditions. Furthermore, we discuss the role of certain sugar-binding proteins, termed galectins, to tune glycoprotein uptake by inducing their crosslinking into lattices, or by co-clustering them with glycolipids into raft-type membrane nanodomains from which the so-called clathrin-independent carriers (CLICs) are formed for glycoprotein internalization into cells. The latter process has been termed glycolipid–lectin (GL-Lect) hypothesis, which operates in a complementary manner to the clathrin pathway and galectin lattices.

## Introduction

Endocytosis is the process by which extracellular or plasma membrane cargoes are internalized in membrane-bounded carriers of different morphologies. Some endocytic processes such as phagocytosis only operate in specialized cell types [1]. In contrast, the so-called micropinocytosis, i.e., endocytic processes that involve tubular or vesicular carriers of sizes of maximally a few hundreds of micrometers, operates in all eukaryotic cells. Micropinocytic uptake processes are categorized into clathrin-dependent [2, 3] and clathrin-independent [4, 5].

Clathrin-dependent endocytosis remains the best characterized pinocytic process [2, 3]. Adaptor proteins such as AP-2, DAB, and Numb recognize signals in the cytosolic tails of cell surface transmembrane proteins and link them to the self-assembly capacity of clathrin to orchestrate the construction of endocytic pits from which clathrin-coated vesicles detach through the pinchase activity of dynamin [6].

Several micropinocytic uptake events continue to operate even when the clathrin pathway is inhibited. These are generically termed clathrin-independent endocytosis processes [4, 5]. The first cargoes for which a non-clathrin mechanism of uptake was suggested were the exogenous bacterial cholera toxin and the plant toxin ricin [7, 8]. Since these early days, several elements of molecular machinery have been identified that contribute to explain how endocytic pits can be built without the need for the clathrin machinery. To name a few, key players that have been particularly well studied are small GTPases [9–11], BAR domain proteins [12, 13], and glycosylation [14]. Caveolae, for a long time portrayed as prototypical clathrin-independent endocytosis carriers, are generally now viewed as mechanosensing, mechanosignaling, and mechanotransduction devices [15].

Clathrin-independent uptake processes have in common that they are particularly sensitive to interference with the activity of the actin cytoskeleton and the organization of the membrane in raft-type nanodomains. According to the most recent understanding, the raft term describes nanodomains in membranes that are inducibly enriched in the so-called raft fabric, i.e., (glyco)sphingolipids, GPI-anchored proteins, other long-chain lipids, cholesterol, and certain transmembrane proteins [16–19]. Among the multitude of possible inducers, 2 are mentioned here as examples: oligomeric glycosphingolipid (GSL)-binding ligands (e.g., the bacterial Shiga and cholera toxins [20]) and the actin-driven molecular focusing of raft components [21]. At some instances in this review, the raft term is used based on older literature in which the association with detergent-resistant membranes was taken as a key indicator for raft nanodomains (see ref. [17] for discussion).

Of note, for some (and possibly most) ligands, clathrin-dependent and clathrin-independent endocytosis may operate in parallel in the same cells. For example, EGFR is internalized by clathrin at low EGF concentrations (below 5 ng/mL), leading to endocytic recycling, while on the same cells at high EGF concentrations (typically above 30 ng/mL), an additional contribution from clathrin-independent endocytosis is measured, leading to lysosomal degradation [22]. An emerging theme from this type of studies is that different forms of endocytic uptake couple to different intracellular distribution schemes, sometimes for the same receptor in the same cells. The molecular mechanisms (ligand concentrations, post-translational modifications, conformational changes…) underlying this complexity often still remain to be elucidated.

Here, we first dissect the mechanism of raft endocytosis of Shiga toxin and expand it to the broader mechanism of the glycolipid–lectin (GL-Lect) hypothesis for clathrin-independent endocytosis driven by sugar-binding proteins (lectins). We then review the literature on the role of protein glycosylation in endocytosis, with an emphasis on clathrin-independent uptake events from raft nanodomain. We point out when functions of corresponding cargoes have been linked to the process of tumorigenesis.

## The Shiga toxin B-subunit as a model of raft endocytosis

Shiga toxin is produced by *Shigella dysenteriae* serotype 1 and by enterohemorrhagic *Escherichia coli* strains [23]. The endocytic uptake of the toxin has been particularly well studied and will be discussed here as a prototypical example of raft endocytosis [20].

**Fig. 1:**
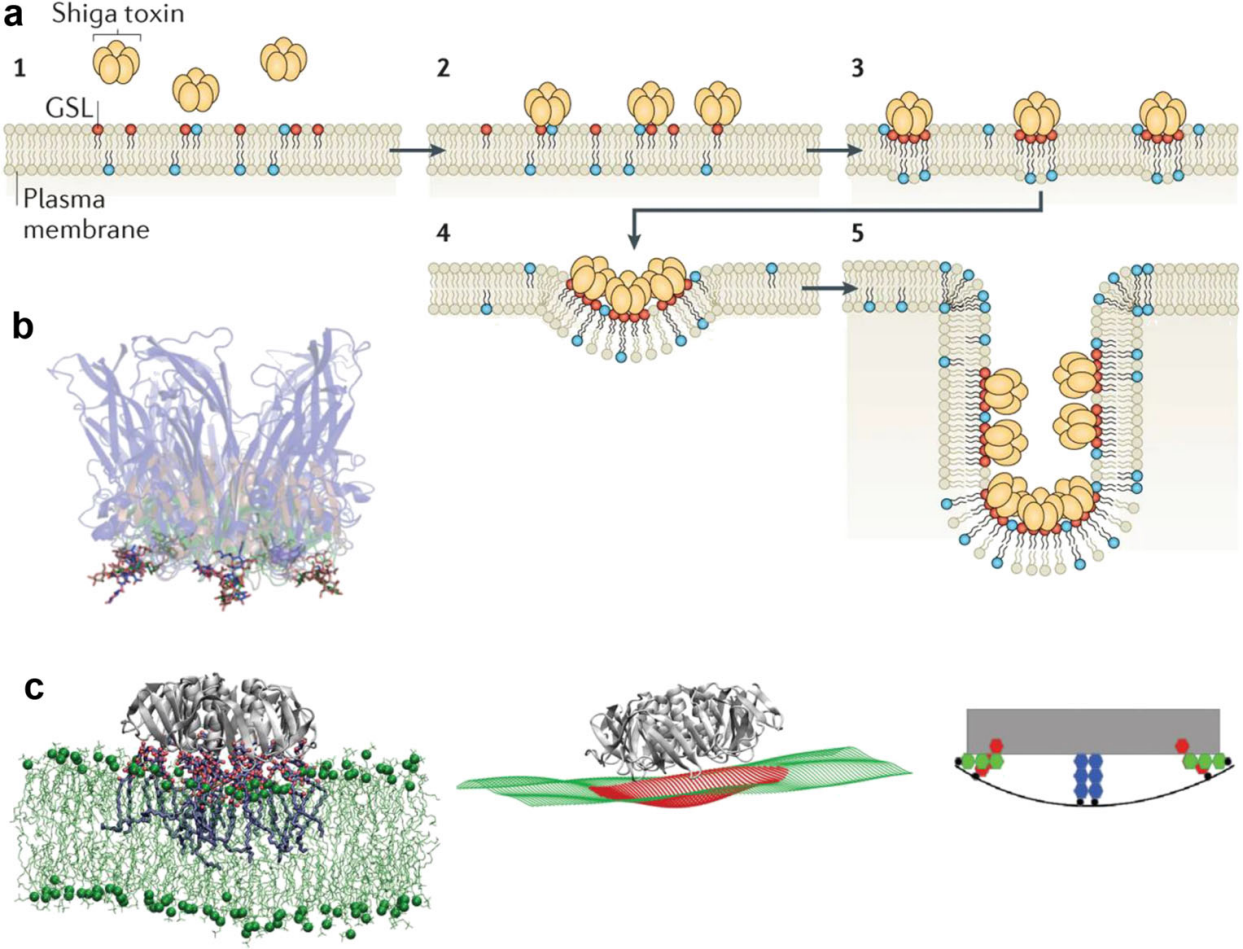
Shiga toxin B-subunit as a model of raft endocytosis. **a** STxB binding to Gb3 induces local membrane curvature, clustering, and the formation of narrow membrane invaginations (reproduced from [4]). **b** Superposition of STxB (green), CTxB (red), and VP1 (blue) structures in interaction with their respective GSL receptors (reproduced from [4]). Note that the conserved binding site 2 positions receptor carbohydrates with similar geometries in space at the rim of the corresponding pathogenic lectins, which is remarkable because the latter do not share any sequence similarity. **c** Molecular dynamics simulation of STxB binding to Gb3 (reproduced from [26]). The binding site geometry with site 3 (blue) under the STxB molecule and sites 2 (green) and 1 (red) at its rim are proposed to imprint an element of negative curvature onto the membrane

Shiga toxin is composed of two parts: a cytotoxic A-subunit and a pentamer of identical B-fragments that form the B-subunit, STxB [23]. STxB binds to the cellular toxin receptor, the GSL globotriaosylceramide (Gb3). Each STxB homopentamer possesses 15 Gb3 binding sites (3 per monomer), that only have millimolar affinity for the globotriose sugar (reviewed in ref. [24]). The high apparent affinity of STxB for cells (in the nanomolar range) is due to multiple bond interactions between each single STxB molecule and several plasma membrane-standing Gb3 molecules at a time [24]. STxB interaction with Gb3 not only serves for toxin recruitment onto target cells. Macroscopically, upon binding to Gb3 on cell or model membranes, STxB induces narrow tubular endocytic pits without the need of the clathrin machinery [25] (Fig. 1a). This activity is shared by the structurally similar cholera toxin B-subunit (CTxB) and simian virus 40 (SV40) capsid protein VP1, in interaction with the GSL GM1 in these cases [27] (Fig. 1b). Based on molecular dynamics simulations and grazing incidence X-ray diffraction studies, it has been argued that the membrane bending activity of STxB is the result of a specific geometry of its binding sites [26] (Fig. 1c) and its lipid compression capacity [28]. To induce narrow membrane invaginations, several STxB molecules must cluster, which appears to be mediated by membrane-mediated mechanisms (ref. [29]; reviewed in ref. [30]), and possibly also by protein–protein interaction [28].

Direct experimental evidence has been provided in model membranes and on cells for the domain-active properties of CTxB [31] and STxB [25, 32, 33]. Molecular dynamics studies have provided *in silico* evidence for STxB-driven clustering of Gb3 lipids under toxin molecules [26] (Fig. 1c). Since GSLs like Gb3 are raft fabric, one might view STxB (and by extension also CTxB and SV40 VP1) as drivers of raft nanodomain construction in relation to endocytic uptake into cells. Raft connectivity (see ref. [16] for a review) might then explain how exogenously added CTxB relocalizes fluorescently labeled GM1 molecules from the plasma membrane to the endoplasmic reticulum [34], and how exogenously added STxB remains detergent-resistant membrane-associated even at the level of the endoplasmic reticulum which it has reached by retrograde trafficking from the plasma membrane [35].

After their endocytic uptake into cells, Shiga and cholera toxins indeed follow the retrograde trafficking route from endosomes to the trans-Golgi network and the endoplasmic reticulum from where the catalytic fragments of their A-subunits are translocated to the cytosol to inhibit protein biosynthesis [36].

## A broader mechanism for raft endocytosis: the GL-Lect hypothesis

In the previous section of this review, we have presented a mechanistic proposal according to which pathogenic lectins (i.e., the bacterial STxB and CTxB, and the VP1 protein of SV40) drive the GSL-dependent construction of endocytic pits. As it will be discussed below, this mechanistic proposal can be extended to a family of cellular lectins, the galectins, with established roles in tumorigenesis [37]. One of these galectins, galectin-3 (Gal3), has been particularly well studied. Various types of cancer show altered levels of Gal3 expression, and the use of Gal3 has been suggested as a diagnostic or prognostic marker in thyroid, gastric, pancreatic, or colorectal cancers [38–40]. In particular, Gal3 has been associated with chemotherapeutic resistance in breast cancer and with tumor cell migration and invasion [40]. Different strategies are investigated to exploit Gal3 as a therapeutic target in cancer therapy, including the use of small molecule inhibitors [39–41].

It has recently been shown that Gal3 has the capacity to induce tubular membrane invaginations on model membranes and in cells [14], similar to what has been described for Shiga toxin, cholera toxin, polyoma, and noroviruses [25, 27, 42]. Of note, this activity is dependent on Gal3 oligomerization and on the presence of gangliosides in the corresponding membranes [14], suggesting that a similar mechanism as for the pathogenic lectins is operating here. Furthermore, Gal3 and another galectin, Gal4 [14], are found in morphological distinct short tubular endocytic carriers, termed clathrin-independent carriers (CLICs), that have previously been described for the cellular uptake of cholera toxin, glycosylphosphatidylinositol (GPI)-anchored proteins, and the cancer stem cell marker CD44 [43, 44], again arguing for strong similarities.

**Fig. 2:**
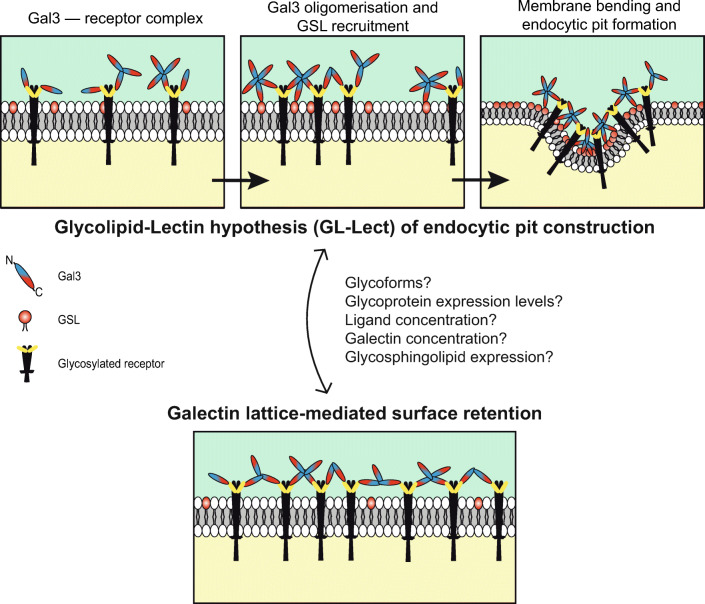
Effect of glycosylation on receptor endocytosis. Schematic representation of 2 alternative scenarios. Top: the GL-Lect hypothesis for the construction of endocytic pits (adapted from [14]). Bottom: galectin lattices for glycoprotein retention at the cell surface. Different conditions are indicated that might allow tuning the equilibrium between both states. The term glycoform comprises for a given glycoprotein the number of glycans and the types of glycans per site (glycan sequence and branching). Gal3, galectin-3; GSL, glycosphingolipid

As opposed to the pathogenic lectins which are their own cargoes, Gal3 drives the clathrin-independent but GSL-dependent endocytic uptake of cellular proteins such as CD44 and β1 integrin [14]. More recently, a similar activity has been described in T lymphocytes for Gal8 and the immunoglobulin superfamily member CD166 [45]. Based on all these studies, a model, termed the GL-Lect hypothesis, has been suggested on how endocytic pit formation might be operated here [46]: Gal3 binds as a monomer to the glycosylated cargo proteins. Upon oligomerization, Gal3 gains the capacity to interact with GSLs in a similar way as described for the pathogenic lectins, leading to the induction of inward-oriented curvature and the formation of tubular endocytic pits from which CLICs detach for the cellular uptake of the cargoes (Fig. 2, top). According to the GL-Lect hypothesis, Gal3 acts like an endocytic adaptor that links glycosylated cargo proteins to the curvature generating device, here: GSLs.

In a previous review, an emphasis was put on GSLs and lectins (notably galectins) and their endocytic functions [46]. In the following, we will therefore focus on glycoproteins and the role that carbohydrate modifications play in their endocytic uptake into cells.

## Endocytosis of cancer-related glycosylated proteins

Cancer cells show altered glycosylation, which plays a key role in cancer development and progression (reviewed in ref. [47]). These alterations can notably occur due to mislocalization or changes in expression of glycosyltransferases, or due to changes in the availability of substrates or cofactors [47]. For instance, GlcNAc (a glucose derivative which is then converted to UDP-GlcNAc by the hexosamine biosynthetic pathway and incorporated into glycans by glycosyltransferases) induces a switch-like increase in the production of tri- and tetra-antennary glycans that galectins bind to [48]. In the context of altered cancer cell metabolism, more UDP-GlcNAc is produced [49], which increases glycan branching and galectin affinity for correspondingly modified glycoproteins. This could impact their endocytosis through either the GL-Lect mechanism (see above) or galectin lattices.

Galectin lattices are cell surface assemblies of glycoproteins that are crosslinked by galectins [50, 51] (Fig. 2, bottom). Evidence for these lattices was first reported in T cells, where both Mgat5 knockdown (the enzyme responsible for synthesis of tetra-antennary glycans that are preferred galectin binding substrates) and galectin inhibition by lactose were shown to enhance TCR recruitment to the site of antigen presentation, as if a galectin lattice on the cell surface was restricting TCR mobility [52]. Mgat5-catalyzed glycan branching and galectins were later shown to play a role in cell surface retention and increased signaling of some key cytokine receptors in cancer [53]. Interestingly, both N-glycan numbers and metabolism regulate receptor retention through galectin lattices: glycoproteins with a low number of N-glycans are ultrasensitive to GlcNAc concentrations and show a switch-like response in surface expression in a narrow range of GlcNAc concentrations, whereas glycoproteins with a high number have a more progressive hyperbolic response to increasing GlcNAc concentrations [48]. Galectin lattices were shown to have pathological importance in autoimmune diseases such as multiple sclerosis [54–56] and in metabolism [57–60].

In this section, we will review cancer-related proteins for which evidence has been presented as to the role of glycosylation in their endocytic uptake into cells. We discuss in each case the endocytic processes that are involved—clathrin-dependent or not—and the evidence for raft nanodomains as substrates for endocytic pit construction.

The GL-Lect hypothesis and galectin lattices provide conceptual frameworks to understand possible links between protein glycosylation and endocytosis (Fig. 2). In the following, special attention will therefore be paid to galectins and to N- and O-glycans on proteins that have been shown to be preferentially recognized by galectins (see ref. [61] and references therein). Apart from primary binding determinants (i.e., β-galactosidic linkages, preferably as N-acetyllactosamine), specific aspects of complex glycan numbers per cargo molecule, sequence, and branching are also of critical importance for galectin interaction with carbohydrates [48]. Furthermore, the balance between cell surface retention through galectin lattices and endocytosis through the GL-Lect mechanism likely also depends directly on the extracellular galectin concentration. CD44 endocytosis for instance is rescued in Gal3-depleted cell by the addition of 0.01 to 1 μg/mL of exogenous Gal3 [14]. At concentrations of 10 μg/mL, however, Gal3 does not rescue CD44 endocytosis [14], likely due to galectin lattice formation. Finally, expression levels of glycolipids are another element to be considered in this context, as endocytic pit construction according to the GL-Lect hypothesis requires the presence of corresponding GSL species.

Galectin lattices and the GL-Lect hypothesis are not necessarily antagonistic and may function cooperatively to dynamically regulate individual glycoprotein levels at the cell surface (Fig. 2). We hypothesize the existence of an equilibrium for individual glycoproteins between endocytosis by the GL-Lect mechanism and cell surface retention within the galectin lattice [62, 63]. The galectin lattices could thus represent a reservoir from which glycoproteins are recruited for clathrin-independent endocytosis upon a modulation of the galectin–glycan interaction strength.

As it was already mentioned in Section 1, many ligands and their receptors are internalized by several endocytic processes on the same cells, which then most likely leads to different intracellular fates. In addition to ligand concentration [22], other factors such as glycan modifications (i.e., glycoforms of the same protein) can be invoked here [64]. In the context of endocytosis, it is therefore of importance to point out that the measure of net cell surface disappearance may not be sufficient to understand endocytic phenotypes. Rather, it is also necessary to test whether the type of endocytosis (clathrin-dependent or not, sugar-dependent or not…) is changed. In other words, while the net uptake of an endocytic cargo may not be altered by a given experimental manipulation, the uptake pathway may well be.

In the following paragraphs, we will also point out experimental aspects that are specific for endocytosis research. *Timing* is one of these. In order to relate experimental outcomes as directly as possible to endocytosis, incubation times of cells with endocytic ligands should be short (typically in the order of 10 min or less). After longer times of incubation, other phenomena such as endocytic recycling or degradation in lysosomes may become dominant, which then complicates the interpretation of experimental findings. Another aspect concerns the *removal of non-internalized ligand by appropriate procedures* (e.g., acid wash, competition with soluble ligands, non-membrane permeable reducing agents), such as to be able to state positively that remaining cell-associated signal comes from truly internalized ligands or transmembrane proteins. Finally, it is important to consider whether an endocytic process is occurring in *a constitutive manner, or whether it is ligand-induced*. In the latter case, the concentration of ligand may be of critical importance for the type of endocytic process that is being triggered.

### Sphingosine-1-phosphate receptor 1

The sphingosine-1-phosphate receptor 1 (S1PR1), also known as endothelial differentiation gene-1 product, is a G protein–coupled receptor which, upon binding of extracellular sphingolipid sphingosine-1-phosphate (S1P), initiates signaling cascades involved in cell survival, cell motility, and angiogenesis. The receptor was shown to promote migration, invasion, and neovascularization in various types of cancer [65]. S1PR1 is glycosylated on its extracellular N-terminal domain at the asparagine 30 position [66]. As assessed by immunofluorescence microscopy in CHO cells, S1PR1 is internalized within 5–10 min upon stimulation with a concentration of 10 nM of S1P, whereas the glycosylation-deficient mutant N30D needs higher concentrations of S1P (≥ 50 nM) to reach similar levels of internalization [66]. Additional evidence of glycosylation increasing ligand-induced internalization was provided upon stimulation with 1 to 50 nM S1P for 3 min followed by washing and proteinase K digestion of cell surface–accessible material at 4 °C. At all tested concentrations of S1P, the percentage of remaining (thus internalized) S1PR1 was decreased for the N30D mutant, compared to wild-type conditions [66].

In HeLa cells, depletion of clathrin heavy chain leads to a strong decrease in S1PR internalization after 30 min incubation with 1 μM of S1P [67]. This S1P concentration is much higher than the ones that were used in the experiments in which S1PR1 glycosylation had an effect on its internalization (1–50 nM), and the incubation of 30 min is much longer than the 5–10 that was used in ref. [66]. It would therefore be interesting to test by which mechanism (clathrin-dependent or not) S1PR1 endocytosis operates at low versus high S1P concentrations, when measured at short times of incubation.

Interestingly, Gal1 has been linked with S1PR1 in gastric cancer: both proteins are overexpressed in gastric cancers, and both are associated with poor prognosis. Gal1 furthermore promotes gastric cancer invasion through a mechanism dependent on S1PR1 overexpression [68]. A direct interaction between glycosylated S1PR1 and Gal1 and a potential link with S1PR1 endocytosis remain to be established.

### Dopamine transporter

The dopamine transporter SLC6A3 was identified as a biomarker for renal cell carcinoma. High expression levels correlate with shorter periods of progression-free survival [69]. The dopamine transporter is glycosylated at three positions [70]. As assessed by confocal microscopy in transfected HEK293 cells, the wild-type dopamine transporter mostly localized at the cell surface, whereas double N181-188Q and triple N181-188-205Q glycosylation-deficient mutants showed significant intracellular localization [70]. Labeling of dopamine transporter at the cell surface with cleavable biotin followed by incubation at 37 °C for 2.5 to 15 min and subsequent stripping of remaining surface-exposed biotin with membrane-impermeable MESNA showed that constitutive endocytosis increased with the number of glycosylation sites that were removed by mutagenesis (N181Q, N181-188Q, N181-188-205Q) [70]. Both clathrin-dependent endocytosis with stimuli such as protein kinase C activation and clathrin-independent endocytosis upon treatment with the small molecule AIM-100 were reported [71–73]. To what extent stimulated endocytosis was glycosylation-dependent remains to be studied.

### Dopamine receptors D2 and D3

Dopamine receptors are a family of five G protein–coupled receptors which have been associated with the regulation of cell death, proliferation, invasion, and migration in different types of tumors [74]. Antipsychotic dopamine receptor antagonists might be interesting treatment strategies against cancer and cancer stem cells [74, 75].

The glycosylation-deficient dopamine D_2_ receptor (D2R) N5-17-23Q showed lower levels of steady-state cell surface localization, as assessed by confocal microscopy and [^3^H]-spiperone and [^3^H]-sulpiride binding studies [76]. The first ligand is hydrophobic and binds both intracellular and cell surface–exposed dopamine D_2_ receptor, whereas the second one is hydrophilic and can only bind cell surface–exposed receptor. Binding studies with [^3^H]-sulpiride after 1 h stimulation with 10 μM dopamine showed increased internalization of glycosylation-deficient D2R, when compared to wild-type [76, 77]. This incubation time is quite long, and it cannot be excluded that other effects than endocytosis also contributed to the observed changes in internalization. Of note, D2R associated with caveolin-1 (Cav1) after 2 min treatment with 10 μM dopamine, as assessed by D2R immunoprecipitation. This interaction was decreased for cells that were treated with the N-glycosylation inhibitor tunicamycin. Depletion of Cav1 increased the remaining cell surface levels of wild-type D2R upon 1 h stimulation with 10 μM dopamine, but did not affect those of glycosylation-deficient D2R [77]. Whether these effects of Cav1 on the cell surface dynamics of D2R were directly related to endocytic uptake was not addressed.

The dopamine D_3_ receptor (D3R) is glycosylated at four potential glycosylation sites, of which two are on the extracellular N terminus (N-12 and N-19) and two are in different extracellular loops (N-97 and N-173) [77]. Individually mutated receptors for each of these positions showed mainly basal surface localization, similar to wild-type. In contrast, the double mutant N12-19Q showed increased intracellular localization, as assessed by confocal microscopy and by [^3^H]-spiperone and [^3^H]-sulpiride binding studies [77]. As opposed to D2R, ligand-induced internalization of D3R (100 nM phorbol myristate acetate for 30 min) was decreased in the case of the glycosylation-deficient mutants N12-19Q, N97-173Q, and N12-19-97-173Q [77]. Since incubations were done for 30 min at 37 °C, care must be taken with ascribing these results purely to endocytic uptake. The association with clathrin heavy chain that was observed with wild-type D3R after 2 min stimulation with 100 nM phorbol myristate acetate was lost on cells that were treated with the N-glycosylation inhibitor tunicamycin (as assessed by D3R immunoprecipitation) [77].

### Glucose transporters 2 and 4

Cancer cells depend on an elevated glucose metabolism. Not surprisingly, facilitative glucose transporters (GLUTs), which funnel glucose along its concentration gradient, are aberrantly expressed in various types of cancer [78].

GLUT2 possesses a conserved single glycosylation site. Deletion of Mgat4a, the gene encoding for N-acetylglucosaminyltransferase IVa essential for the biosynthesis of tri- and tetra- antennary glycans, leads to the relocalization of GLUT2 from the cell surface to intracellular compartments, as assessed by flow cytometry and immunofluorescence microscopy on pancreatic cells from Mgat4a^−/−^ mice [79]. Pulse-chase analysis of newly synthesized GLUT2 with [^35^S]methionine showed no difference in arrival to the cell surface between Mgat4a^−/−^ and control cells. The degradation of cell surface biotinylated GLUT2 was accelerated in Mgat4a^−/−^ cells upon incubation of 3–15 h [79]. Unfortunately, no acute endocytosis study was performed. Interestingly, GLUT2 and Gal9 colocalized and coimmunoprecipitated in normal cells, but not in Mgat4a^−/−^ cells [79]. N-glycosylation possesses an additional layer of regulation of GLUT2: deletion of Mgat4a redistributed GLUT2 to lipid raft nanodomains and attenuated its activity, which was regained upon raft disruption by treatment with methyl-β-cyclodextrin [80].

GLUT4 also possesses a single glycosylation site. Supplementing HEK23T cells with increasing concentrations of GlcNAc induced a switch-like response by increasing the percentage of cell surface GLUT4, as one would expect if GLUT4 was retained at the cell surface in a glycosylation and galectin-dependent manner. Mutation of the glycosylation site abolished this response [48].

### Epidermal growth factor receptor

The epidermal growth factor receptor (EGFR) is a tyrosine kinase receptor, which, upon ligand binding, activates proliferation and survival pathways [81]. EGFR is often mutated or overexpressed in carcinoma patients [81]. EGFR is N-glycosylated on 8 of the 11 canonical putative sites [82, 83].

Depletion of Mgat5 decreased EGFR ligand-induced activation [84], downstream signaling, and tumor cell invasiveness–related phenotypes [85]. Knockout of Mgat5 was furthermore shown to lower the binding of cell surface EGFR with Gal3, and increased constitutive EGFR colocalization with endosomes [53]. Both lactose treatment and Mgat5 knockout, which disrupt galectin lattices, increased the association of EGFR with Cav1, which suppressed EGFR signaling [86].

Moreover, Mgat5 depletion was shown to inhibit ligand-induced degradation/downregulation of EGFR [87]. At the high EGF concentrations as those that were used in this study (100 ng/mL), EGFR is known to be internalized both by clathrin-dependent and clathrin-independent endocytosis, with the former preferentially targeting EGFR for recycling to the plasma membrane, while the latter preferentially targets EGFR to degradation [88]. A possible explanation for inhibition of EGFR downregulation by Mgat5 depletion is that clathrin-independent EGFR endocytosis is inhibited under these conditions. Indeed, it was found that after EGF binding at 4 °C followed by incubation for 5–15 min at 37 °C, EGFR was less efficiently internalized in Mgat5-depleted cells, when compared to control cells [87]. This was shown using two different methods: (1) stripping, trichloroacetic precipitation, and western blot detection of the remaining surface-bound EGF, and (2) labeling with biotin of the remaining cell surface proteins and isolation of these by streptavidin pull down, followed by EGFR western blot detection [87].

Studies on the effect of glycosylation on EGFR activity and trafficking used methods modulating glycosylation globally. The effects found on EGFR localization or signaling might thus be indirect. For instance, Gal3 interacts with the cell surface glycoprotein MUC1, leading to altered cell surface expression of MUC1 and enhanced MUC1–EGFR association, which increases EGFR activation [89]. Mgat5 depletion most likely impacts MUC1 glycosylation, which might then affect EGFR. It would therefore be important to study glycosylation-deficient mutants of EGFR and to test their endocytosis via clathrin-dependent or clathrin-independent uptake mechanisms [88, 90].

Targeting of glycans might be an interesting strategy to modulate EGFR activity in cancer. 1,3,4-O-Bu3ManNAc, which increases overall sialylation by 2 fold, and EGFR sialylation in particular by ~ 20–30%, decreased EGFR activation and synergized with the tyrosine kinase inhibitor drugs erlotinib and gefitinib, resulting in re-sensitization of resistant cells to these treatments [91, 92]. 1,3,4-O-Bu3ManNAc weakened the galectin lattices and increased EGFR internalization, mainly through clathrin-independent endocytosis [92].

### Vascular endothelial growth factor receptors

Vascular endothelial growth factor receptors (VEGFRs) play a key role in angiogenesis and are often highly expressed in cancers [93]. Extracellular VEGFR2, which possesses 18 putative N-glycosylation sites, interacted in a glycosylation-dependent manner with Gal3, as shown by coimmunoprecipitation, and depletion of Mgat5 largely abolished this interaction [94]. The impact of Gal3 and Mgat5 on ligand-induced VEGFR2 endocytosis was assessed in Gal3- and Mgat5-depleted cells. For this, cell surface proteins were labeled with cleavable biotin, cells were incubated for 5–20 min at 37 °C with 80 ng/mL VEGF-A, the remaining surface-exposed biotin was removed, cells were lysed, and internalized proteins were isolated using streptavidin beads [94]. Depletion of Gal3 or Mgat5 increased ligand-induced VEGFR2 internalization after 5–10 min, with Mgat5 depletion having the greatest impact. Both depletions were shown to reduce angiogenesis [94]. Exogenous addition of 1 μg/mL of Gal1 and/or Gal3 and incubation for as long as 2 h decreased the colocalization between VEGFR1 or VEGFR2 and early endosome antigen-1 [95], which because of the long incubation period could have been due to several intracellular events such as reduced recycling and/or reduced targeting to the late endocytic pathway, in addition to a possible effect on endocytic uptake. The two receptors had different sensitivities to galectin modulation: exogenous addition of Gal1 or Gal3 was sufficient to enhance VEGFR2 phosphorylation, whereas VEGFR1 required both galectins [95].

A specific VEGFR2 glycosylation-deficient mutant N247Q increases receptor activation, dimerization, and degradation, with no significant change in ligand-induced internalization after 10 min as assessed by a cell surface biotinylation strategy [96]. Surface VEGFR2 has more complex glycans than intracellular VEGFR2, with sialylation notably at the N247 site. Neuraminidase treatment, which removes sialylation, increases WT VEGFR2 activation at levels similar to N247Q [96]. N247 is in the kinase site and its sialylation might directly hinder dimerization [96], on top of galectin-mediated effects of VEGFR2 glycosylation presented above.

Interestingly, the vasculature of tumors that are sensitive to anti-VEGF treatment showed increased sialylation, which prevents Gal1 binding. On the contrary, vessels of tumors resistant to anti-VEGF treatment had a glycosylation pattern that facilitated Gal1 binding, and resistant tumors secreted more Gal1 [97]. Depletion of Gal1 restored sensitivity to anti-VEGF treatment in these tumors [97]. Thus, modulating glycosylation pattern of tumor vasculature or galectin concentration might increase the efficacy of anti-VEGF treatment.

### Fibroblast growth factor receptor

Fibroblast growth factor receptors (FGFR) are a family of tyrosine kinase receptors that activate major survival and proliferation pathways. They have been implicated in a wide range of cancers [98]. FGFR can be glycosylated at several extracellular positions, and glycosylation may affect FGF binding [99, 100]. Mgat5 knockout drastically decreased FGF signaling [53]. FGF signaling in Mgat5 knockout cells hyperbolically increased upon supplementation with increasing GlcNAc concentrations, as for EGFR which also has a high number of glycosylation sites [48]. This is at the opposite to TGF-β receptors, CTLA-4 and GLUT4, which have fewer glycosylation sites and showed a switch-like response to increasing GlcNAc concentrations [48].

Interestingly, Gal1 and Gal3 have been shown to bind the extracellular domain of all FGFRs [101]. Gal3 had higher affinity for FGFR1 than Gal1 and competed for binding [101]. The two galectins had different impact on basal FGFR1: Gal1 promoted constitutive activation of FGFR1, whereas Gal3 inhibited constitutive FGFR1 internalization [101].

### Death receptors 4 and 5 (TRAIL receptors)

Upon binding to the ligand tumor necrosis factor-related apoptosis-inducing ligand (TRAIL), death receptors 4 and 5 trimerize, leading to the formation of the death-inducing signaling complex (DISC) and apoptosis induction, notably in cancer cells. TRAIL might thus be an interesting molecule to treat cancer pathologies, even if some resistance issues still need to be overcome [102]. The influence of glycosylation on death receptors (DR) 4 and 5 is reviewed in depth in [103]. Briefly, DR5 was shown to be O-glycosylated, and mutations of the putative O-glycosylation sites did not impact TRAIL binding, but reduced its ability to induce apoptosis [104]. Mutation of the N-glycosylation sites N99-122A of mouse DR4 increased internalization of the receptor, as assessed with differential immunolabeling between cell surface and internalized DR4 (wild-type or glycosylation mutants) after TRAIL stimulation [105]. Mutation of the unique N-glycosylation site of human DR4 N156A lowered receptor aggregation and DISC formation and reduced apoptosis induction [105].

Interestingly, a TRAIL-resistant cell line was obtained and studied. It possessed increased Gal3 levels and showed reduced TRAIL-induced DR4 and DR5 internalization, as assessed in immunofluorescence experiments by pre-labeling of cells with antibodies and subsequent stimulation by incubation with 100 ng/mL TRAIL for 30 min at 37 °C, followed by acid wash to remove the remaining surface-accessible antibodies. Inhibitors of Gal3 binding or inhibitors of glycosylation re-sensitized this cell line to TRAIL [106].

### Discoidin domain receptor 1

The binding of discoidin domain receptor 1 to collagen triggers signaling pathways that are critical for cell–collagen interaction and collagen remodeling. Discoidin domain receptor 1 was shown to play an important role in cancer progression [107]. The receptor possesses 4 putative N-glycosylation sites (2 confirmed) and 2 potential O-glycosylation sites [108]. Mutants in which single N-glycosylation sites were removed showed ligand-dependent phosphorylation, as observed for the wild-type receptor, except N211Q which was constitutively phosphorylated. Interestingly, while wild-type receptor showed ligand-induced internalization, internalization of N211Q did not increase in the presence of collagen [108]. This result was obtained by pre-treating or not cells expressing wild-type or N211Q discoidin domain receptors with 10 μg/mL collagen I for 30 min at 37 °C, followed by labeling of cell surface proteins on ice with cleavable biotin and incubation for 0–40 min at 37 °C, cleavage of biotin that had remained cell surface–exposed, and isolation of internalized proteins with streptavidin beads.

### β2-adrenergic receptor

The β2-adrenergic receptor is a G protein–coupled receptor for epinephrine. It has been associated with development and progression of different types of cancer [109, 110]. The β2-adrenergic receptor possesses two glycosylation sites in the N-terminal domain (N6 and N15) and one in an extracellular loop (N187) [111]. Glycosylation-deficient mutants N6Q, N15Q, and N6-15Q, but not N187Q, had an increased isoproterenol EC50, meaning less effective β2-adrenergic receptor activation [111]. They furthermore showed decreased receptor dimerization and ligand-induced internalization, which was quantified as the loss of cell surface receptors measured by flow cytometry after 30 min stimulation with 10 μM isoproterenol [111]. Decreased receptor dimerization in N6Q, N15Q, and N6-15Q mutants was likely responsible for decreased internalization since other mutations decreasing β2-adrenergic receptor dimerization (K60A-E338A) also showed decreased isoprotenerol-induced internalization, as measured by the same method [111].

### The glutamine transporter ASCT2 (SLC1A5)

The glutamine transporter ASCT2 is often overexpressed in different types of cancer and associated with bad prognosis [112]. Cancer cells indeed often use glutamine metabolism for energy production and as biological material for sustained growth and proliferation [112]. ASCT2 possesses 2 glycosylation sites. The N-glycosylation–deficient mutant N163-212Q showed delayed trafficking of newly synthesized proteins to the plasma membrane, decreased cell surface localization at steady state, enhanced internalization (as assessed by reversible cell surface biotinylation and biotin stripping after 15, 30, or 60 min incubation at 37 °C), and decreased stability, but no difference in functionality [113]. Of note, Gal12 was shown to bind ASCT2 and reduced glutamine uptake [114]. The impact of Gal12 or other galectins on ASCT2 localization or endocytosis was not studied, however.

### CD44

CD44 is a cell adhesion molecule which has also been implicated in the regulation of growth, survival, differentiation, and motility [115]. CD44 overexpression or alternative splicing was described for many types of cancers [116]. CD44 is a known cargo of clathrin-independent endocytosis [117]. CD44 possesses 5 putative N and 7 putative O-glycosylation sites [118, 119]. A N-glycosylation–deficient mutant of CD44 failed to be efficiently internalized, as evaluated in anti-CD44 antibody uptake experiments after incubation for 10 min at 37 °C and acid wash to remove remaining cell surface–exposed antibody [14]. CD44 endocytosis was also dependent on GSLs and Gal3, which provided first evidence for GL-Lect endocytosis of an endogenous cargo [14]. CD44 uptake was rescued in Gal3-depleted cells with as little at 10 ng/mL (a concentration that is similar to the ones found in human serum; see ref. [14] and references therein) up to 1 μg/mL of exogenously added Gal3. In a recent study, the GSL- and Gal8-dependent endocytic uptake of CD166 in lymphocytes could also be rescued with such low concentrations of Gal8 [45]. Ten μg/mL of exogenously added Gal3 did not rescue CD44 uptake [14], likely due to cell surface retention at this high concentration.

### α5β1 Integrin

Integrins are cell adhesion molecules that recognize components of the extracellular matrix, and that are involved in cancer initiation, proliferation, migration, and metastasis [120]. Integrins form a family of 24 heterodimers generated from a combination of 18 α- and 8 β-subunits [120]. A well-studied member, α5β1 integrin, possesses 26 potential N-linked glycosylation sites, 14 in the α5-subunit and 12 in the β1-subunit [121].

Several glycosylation–deficient mutants of β1 integrin have altered surface expression levels, when compared to wild-type protein [121]. No direct comparison of acute uptake rates was performed, making it difficult to conclude on the contribution of glycosylation to endocytosis. The glycosylation sites 4 to 6 are necessary for α5β1 heterodimer formation [121].

An α5 integrin glycosylation–deficient mutant, containing only the N-glycosylation sites 3–5, showed increased cell surface localization and delayed internalization of the active conformation of α5β1 integrin [122]. Endocytosis was measured by incubation for 2.5 or 5 min at 37 °C, using a reversible cell surface biotinylation strategy. At the cellular level, this mutant caused increased cell-matrix adhesion and decreased migration [122]. Interestingly, generation of additional glycosylation-deficient mutants identified N-glycosylation sites 1 and 2 as being mainly responsible for these effects [122].

β1 integrin activity has been linked with the expression of several gangliosides such as GT1b [123], GD3 [124], or GM2 [125]. β1 integrin has also been shown to interact with Gal1 [126], Gal3 [14], and Gal8 [127]. Gal1 and β1 integrin are related in several types of cancer: treatment with 50 to 200 μg/mL Gal1 inhibits growth of epithelial tumor cell lines, and this effect is greatly diminished in the presence of an α5β1 blocking antibody [128]. Gal1 knockdown in glioma cell lines leads to the intracellular accumulation of β1 integrin [129] and decreases cell motility [130]. Cancer-associated fibroblasts expressing high Gal1 levels in co-culture with gastric cancer cells increase their migration and invasion. This effect is canceled upon cancer-associated fibroblast treatment with Gal1 siRNA or upon gastric cancer cell treatment with β1 integrin siRNA, showing both proteins’ involvement in the process [131]. Expression of both Gal1 and β1 integrin in gastric cancer patients leads to poor prognosis [131].

Gal3 was shown to regulate cell migration on fibronectin and fibronectin fibrillogenesis, which depends on the active conformation of α5β1 integrin [132]. Interestingly, both processes were maximally enhanced at 1–2 μg/mL of Gal3, whereas this stimulatory effect was lost at a concentration of 5 μg/mL [132]. Gal3 plays a crucial role in β1 integrin endocytosis: β1 integrin internalization as detected after 10 min incubation at 37 °C in an antibody uptake experiment was shown to be dependent on Gal3, and Gal3 colocalized with β1 integrin in tubular structures [14]. Both clathrin-dependent and clathrin-independent endocytosis of β1 integrin have been reported [133, 134], and the GL-Lect hypothesis likely explains the clathrin-independent part. Interestingly, a positive feedback loop was described between Gal3 and β1 integrin, with β1 integrin stimulating the epigenetic activation of Gal3 transcription, and Gal3 promoting β1-mediated cell adhesion and migration [135]. Single particle tracking showed that Gal3 influences the lateral mobility of α5β1 integrin in HeLa cells and increases α5 integrin cluster formation and cell migration [136].

### E-cadherin

Cadherins are transmembrane proteins mediating calcium-dependent cell–cell adhesions. The loss of E-cadherin is a key marker of epithelial to mesenchymal transition in cancer cells [137]. In canine mammary gland models, the glycosylation profile of E-cadherin differs between adenomas and carcinomas, with increased glycan branching and sialylation in carcinomas [138]. Mgat3 (which generates bisecting GlcNAc structure) and Mgat5 (which induces tetra-antenna glycan branching) differentially regulate E-cadherin: Mgat3 overexpression does not affect E-cadherin localization, but increases its total expression levels through delayed degradation, and increases cell–cell adhesion, whereas Mgat5 overexpression enhances the intracellular localization of E-cadherin and decreases cell–cell adhesion [139]. E-cadherin possesses 4 potential glycosylation sites (N554, 566, 618, 633) [140]. N554Q localization at the cell surface is not altered, whereas the triple mutant N566-618-633Q shows increased intracellular localization, a phenotype that is rescued upon Mgat5 depletion [141]. N554Q mutation furthermore induces increased cis-dimerization of E-cadherin and cell–cell aggregation compared to WT or N566-618-633Q [141]. Mgat5 glycan branching also alters the cis-dimerization and functionality of another cadherin protein, the N-cadherin, without affecting its surface localization [142, 143].

Depletion of Gal7, which unexpectedly binds to E-cadherin independently of its glycosylation, increases E-cadherin internalization, as assessed by an antibody uptake experiment for 15, 30, 60, and 120 min, followed by acid washes. As little as 0.01 μg/mL of exogenously added Gal7 restores normal internalization levels, while increased concentrations of exogenously added Gal7 further decrease E-cadherin internalization [144]. Gal7 depletion furthermore increases E-cadherin mobility and decreases E-cadherin–mediated cell–cell adhesion [144].

### Cytotoxic T lymphocyte antigen 4

The cytotoxic T Lymphocyte Antigen 4 (CTLA-4) is an immune checkpoint molecule which downregulates T cell activation. CTLA-4 is targeted in immunotherapy in order to stimulate an immune response toward the tumor [145]. CTLA-4 possesses 2 N-glycosylation sites. At low levels of T cell activation, Mgat5^−/−^ T cells show decreased surface expression of CTLA-4 compared to Mgat5^+/+^ T cells [48]. Similarly, CTLA-4 surface expression is decreased in cells treated with lactose, which competes for galectin binding [48]. A common polymorphism of CTLA-4 leading to its incomplete glycosylation with only one glycosylated site also leads to decreased cell surface localization [146]. As expected, due to the low number of glycosylation sites on CTLA-4 and due to the ultrasensitivity of glycan branching to GlcNAc concentrations, CTLA-4 surface expression increases in a switch-like response to increasing GlcNAc concentrations [48]. CTLA-4 was reported to be constitutively internalized by clathrin-mediated endocytosis, even during T cell activation, and mainly recycled back to the plasma membrane [147].

### Interferon-γ receptor

Interferon-γ (IFN-γ) is a cytokine which induces signaling related to host defense and immune regulation [148]. Depending on the tumor specificity and its microenvironment, IFN-γ has anti-tumorigenic or pro-tumorigenic effects [148]. In the case of IFN-γ receptor, it is not the loss but the gain of a N-glycosylation site which has pointed to a role of glycosylation in the function of this cancer-related receptor. The IFN-γR2 T168N gain-of-N-glycosylation mutation resulted in complete JAK/STAT signaling deficiency [149]. Ligand-induced internalization of IFN-γR2 T168N was studied with radiolabeled ^125^I-IFN-γ, which was bound to cells on ice, upon which these were shifted for 5–40 min to 37 °C, followed by acid washes to remove the remaining surface-bound ^125^I-IFN-γ. The effect of IFN-γR2 T168N mutation on ^125^I-IFN-γ uptake was found to be minor [149]. Instead, it was shown that binding of IFN-γR2 T168N to Gal1 and Gal3 restricted its lateral diffusion to actin nanodomains, which altered its signaling [149].

### Major histocompatibility complex I

The major histocompatibility complex class I (MHCI) molecules are responsible for presenting intracellular antigens to T lymphocytes. They are often downregulated in cancer cells as a way to escape the immune response toward the tumor [150]. MHCI molecules are known cargoes of clathrin-independent endocytosis [117]. They possess a single site of N-glycosylation [151]. Treatment of HeLa cells for 48 h with 10 mM GlcNAc or the addition of 1 or 10 μg/mL exogenous Gal3 led to an increase of anti-MHCI antibody uptake, when the latter was incubated with the cells for 30 min at 37 °C, followed by acid wash to remove the remaining surface-exposed antibody [62]. Lactose treatment, which competes for galectin binding, or Gal3 knockdown abolished the increase linked to GlcNAc complementation [62].

### CD59

CD59 is a GPI-anchored protein that inhibits the formation of the membrane attack complex resulting from complement activation. It is often dysregulated in cancers and could be an interesting target for immunotherapy [152]. CD59 possesses 2 N-glycosylation sites. Contrary to MHCI (see above), 48 h treatment of HeLa cells with 10 mM GlcNAc or the addition of 1 or 10 μg/mL of exogenous Gal3 led to a decrease in CD59 antibody internalization (30 min incubation) [62]. Gal3 depletion increased CD59 antibody internalization [62]. Inhibition of all galectin–glycan interactions by lactose however inhibited internalization of CD59, showing that some galectin–glycan interaction is necessary for its clathrin-independent endocytosis [62]. In contrast to HeLa cells, in human bronchial epithelial Beas2b cells, GlcNAc treatment enhanced CD59 antibody internalization, showing the importance of the cellular context to the effect that glycosylation has on endocytosis [62].

### Neuropilin-1

As a co-receptor, neuropilin-1 modulates the activity of various ligands such as VEGF, TGF-β, HGF, or semaphorins [153], which promotes cancer growth, angiogenesis, and metastasis in various types of cancer [153]. Neuropilin-1 possesses 5 putative N-glycosylations sites [154]. Two splice variants of neuropilin-1 affect the number of glycosylation sites on the protein: NRP1-ΔE4 missing exon 4 lacks the N-glycosylation site N150, and NRP1-ΔE5 missing the exon 5 lacks the N-glycosylation site N261 [154]. Both splice variants were identified in colorectal cancer, with NRP1-ΔE4 expression correlating with cancer progression [154]. These two splice variants were internalized more efficiently upon incubation for 15 min at 37 °C, as assessed using reversible cell surface biotinylation, and displayed increased recycling compared to wild-type NRP1. Furthermore, both splice variants escaped degradation [154]. N-glycosylation–deficient mutants N150Q, N261Q, and N150-261Q showed increased colocalization with the endosomal markers EEA1 and Rab7 and promoted cell migration and invasion [154]. Internalization was not directly measured, however, neither on these N-glycosylation mutants, nor on additional ones that were described in this study (i.e., N300Q, N522Q, and N842Q).

### CCR7

C-C Chemokine Receptor 7 (CCR7) plays an important role in the migration of T cells and dendritic cells to lymph nodes. In cancer cells, CCR7 activation can promote cell migration and metastasis [155]. CCR7 is glycosylated on N36 and N292 [156]. Mutation of these residues enhanced CCL19 and CCL21 signaling and increased cell migration [156]. Mutation N36A, but not N292A, decreased ligand-induced internalization, as assessed by 30 min incubation at 37 °C with CCL19, followed by microscopy analysis of the number of intracellular CCR7-containing structures per cell [156].

### Neurokinin 1 receptor

The neurokinin 1 receptor is a G protein–coupled receptor that is expressed in the central and peripheral nervous system where it activates Ras/Raf/MAPK and/or PI3K/Akt/mTOR signaling pathways. The receptor and its ligand are overexpressed in various types of cancers, and antagonists of the receptor have shown some antitumor effect [157]. The neurokinin-1 receptor is glycosylated at two positions [158]. After pre-incubation with its radiolabeled ligand at 4 °C, incubation for 5 min at 37 °C followed by acid wash to remove the remaining surface-exposed ligand, cells expressing the glycosylation-deficient mutant N14-18Q showed a 24% increase in the internalization of the receptor, compared to cells expressing the wild-type receptor [158].

## Conclusion

From the examples that were discussed above and listed in Table 1, it is apparent that glycosylation modulates the endocytic uptake of a number of proteins with important functions in tumorigenesis. It can therefore be expected that the established impact of glycosylation on cancer is at least in part linked to tuning the cell surface dynamics of these proteins. In most cases, it still needs to be addressed whether interfering with defined glycans on given cargoes leads to their reshuffling between endocytic processes (dependent or not on clathrin). For example, if the overall rate of endocytic uptake of a cargo protein is stimulated upon interfering with its glycosylation, 2 alternative interpretations can be envisaged: its glycosylation-dependent cell surface retention under unperturbed conditions, or its reshuffling from a slower to a faster uptake pathway under glycan perturbation conditions. Similar to this, many other important questions remain to be addressed, such as: Are galectin lattices in dynamic equilibrium with GL-Lect-based construction of endocytic pits, in a way similar to the dynamic link between flat clathrin lattices and clathrin-coated pits? Are all glycan sites on cargo proteins equal as to being used for tuning the cell surface dynamics of a given cargo protein? Can glycan-based mechanisms for the regulation of a cargo protein’s cell surface dynamics be acutely tuned? We expect that the investigation of these and many other questions will provide a fruitful ground for discovery in the field of cellular glycobiology.

**Table 1:** Effect of glycosylation and galectins on the endocytosis of cargo proteins

Protein	Method used to affect glycosylation *(specific or global)*	Ligand-induced or constitutive	Incubation time	Cell surface removal method	Effect of glycosylation mutants/modulation	Comments/other GL-Lect components
Sphingosine-1-phosphate receptor 1 (S1PR1)	*Specific* Mutant N30D	Constitutive (no S1P)	3 min	Proteinase K digestion of cell surface–accessible material at 4 °C	Decreased internalization of N30D compared to WT in both ligand-induced and constitutive internalization [66]	Gal1 has been linked with S1PR1 in gastric cancer [68]
		Ligand-induced (1 to 50 nM S1P)				
Dopamine transporter SLC6A	*Specific* Mutants N181Q, N181-188Q, and N181-188-205Q	Constitutive	2.5 to 15 min	Reversible cell surface biotinylation	Increased internalization with the removal of glycosylation sites [70]	
Dopamine D_2_ receptor	*Specific* Mutant N5-17-23Q	Constitutive	n/a	/	Lower cell surface localization of N5-17-23Q compared to WT [76, 77]	
		Ligand-induced (10 μM dopamine)	1 h	n/a (measure of remaining cell surface material)	Increased internalization of N5-17-23Q compared to WT [76, 77]	Long incubation time, it cannot be excluded that other effects than endocytosis also contributed to the observed changes
Dopamine D_3_ receptor	*Specific* Mutants N12Q, N19Q, N97Q, N173Q, N12-19Q, N97-173Q, and N12-19-97-173Q	Constitutive	n/a	/	Increased intracellular localization of N12-19Q compared to WT and single mutants [77]	
		Ligand-induced (100 nM phorbol myristate acetate)	30 min	n/a (measure of remaining cell surface material)	Decreased internalization of N12-19Q, N97-173Q, and N12-19-97-173Q compared to WT [77]	Long incubation time, it cannot be excluded that other effects than endocytosis also contributed to the observed changes
Glucose transporter 2 (GLUT2)	*Global* Mgat4a deletion (gene encoding for N-acetylglucosaminyltransferase IVa)	Constitutive	n/a	/	Increased intracellular localization of GLUT2 and accelerated degradation of cell surface GLUT2 in Mgat4a^−/−^ cells compared to normal cells [79]	GLUT2 and Gal9 colocalized and coimmunoprecipitated in normal cells, but not in Mgat4a^−/−^ cells [79]
Glucose transporter 4 (GLUT4)	*Global* Supplementation with GlcNAc (increased glycan branching)	Constitutive	n/a	/	Increased cell surface GLUT4 with increasing concentrations of GlcNAc in a switch-like response [48]	
Epidermal growth factor receptor (EGFR)	*Global* Depletion or deletion of Mgat5	Constitutive	n/a	/	Lower cell surface levels and increased colocalization of EGFR with endosomes in Mgat5^−/−^ cells, compared to normal cells [53]	Lower binding of Gal3 to EGFR in Mgat5^−/−^ cells compared to normal cells [53] Increased association of EGFR with Cav1, which suppresses EGFR signaling, in Mgat5^−/−^ cells compared to normal cells [86]
		Ligand-induced (100 ng/mL EGF)	0, 5, 10, and 15 min	n/a (measure of remaining cell surface material)	Decreased ligand-induced internalization of EGFR in Mgat5-depleted cells, compared to normal cells [87]	Mgat5 depletion was shown to inhibit ligand-induced degradation of EGFR [87]
Vascular endothelial growth factor receptor 2 (VEGFR2)	*Global* Depletion of Mgat5	Ligand-induced (80 ng/mL VEGF-A)	0, 5, 10, and 20 min	Reversible cell surface biotinylation	Increased internalization of VEGFR2 after 5 and 10 min in Mgat5-depleted cells [94]	Similarly, increased internalization of VEGFR2 in Gal3-depleted cells [94]
FGFR	*Global* Deletion of Mgat5 ± supplementation with GlcNAc	Ligand-induced	10 min	n/a	Decreased FGF signaling in Mgat5^−/−^ compared to normal cells. FGF signaling in Mgat5^−/−^ cells hyperbolically increases upon supplementation with increasing concentrations of GlcNAc [48, 53]	Gal1 promotes constitutive activation of FGFR1, whereas Gal3 inhibits constitutive FGFR1 internalization [101]
Discoidin domain receptor 1	*Specific* N211Q mutant (out of several single N-glycosylation site mutants, only mutant studied for internalization)	Constitutive and ligand-induced (10 μg/mL rat tail collagen I)	0, 5, 15, 40 min	Reversible cell surface biotinylation	While wild-type receptor showed increased internalization in the presence of the ligand, internalization of N211Q did not increase in the presence of collagen. Constitutive internalization was similar between N211Q and WT receptor [108]	
β2-Adrenergic receptor	*Specific* Mutants N6Q, N15Q, N187Q, and N6-15Q	Ligand-induced (10 μM isoproterenol)	30 min	n/a (measure of remaining cell surface material)	Decreased internalization of N6Q, N15Q, and N6-15Q compared to WT receptor [111]	N6Q, N15Q, and N6-15Q show decreased receptor dimerization. Other mutations decreasing receptor dimerization also decreased internalization in the same conditions [111]. The decreased dimerization might thus explain the decreased internalization here
Glutamine transporter ASCT2 (SLC1A5)	*Specific* Mutants N212Q, N163Q, and N163-212Q	Constitutive	15, 30, 60 min	Reversible cell surface biotinylation	Increased internalization of N212Q and N163-212Q, compared to WT [113]	Gal12 was shown to bind ASCT2 and reduces glutamine uptake [114]
CD44	*Specific* Mutant N25-57-100-110-120S	Constitutive	10 min	Acid wash	Decreased internalization of N-glycosylation–deficient mutant, compared to WT [14]	CD44 endocytosis was also dependent on GSLs and Gal3 [14]
α5β1 Integrin	*Specific* β1 integrin mutants S1–3 (only first 3 N-glycosylation sites kept, other sites changed to Q) and similarly S4–6, S7–8, and S9–12	Constitutive	n/a	n/a	Decreased surface expression of the mutants compared to the WT [121]	β1 integrin internalization following 10 min incubation at 37 °C with anti-β1 integrin antibody was shown to be dependent on Gal3, and Gal3 colocalized with β1 integrin in tubular structures [14]
	*Specific* α5 mutant containing out of the 14 potential N-glycosylation sites only glycosylation sites 3–5 (necessary for heterodimer formation [121])	Constitutive	0, 2.5, 5, 7.5, 10, 12.5, and 15 min	Reversible cell surface biotinylation	Delayed internalization of the active conformation of mutant α5β1 integrin compared to WT [122]. Generation of additional glycosylation-deficient mutants identified N-glycosylation sites 1 and 2 as being mainly responsible for these effects [122]	
E-cadherin	*Global* Mgat3 or Mgat5 overexpression	Constitutive	n/a	n/a	Mgat3 induces delayed degradation and increased total expression of E-cadherin, whereas Mgat5 overexpression increases E-cadherin intracellular localization [139]	
	*Specific* N554Q and N566-618-633Q mutants	Constitutive	n/a	n/a	N566-618-633Q shows increased intracellular localization compared to N554Q and WT E-cadherin [141]	
CTLA-4	*Global* Mgat5 deletion or supplementation with GlcNAc	Constitutive	n/a	n/a	Mgat5 deletion leads to decreased CTLA-4 cell surface expression [48]. CTLA-4 cell surface expression increases in a switch-like response to increasing GlcNAc concentrations [48]	Lactose treatment, which competes for galectin binding, decreases CTLA-4 surface expression [48]
IFN-γ receptor 2	*Specific* Gain-of-N-glycosylation mutant T168N	Ligand-induced (1000 U/mL ^125^I-IFN-γ)	0, 5, 10, 15, 20, 30, 40 min	Acid wash	Similar rate and extent of internalization between T168N and WT receptor [149]	Binding of IFN-γR2 T168N to Gal1 and Gal3 restricted its lateral diffusion to actin nanodomains, which altered its signaling [149]
MHC I	*Global* Supplementation with GlcNAc (increased glycan branching)	Constitutive	30 min	Acid wash	Increased internalization upon supplementation with GlcNAc [62]	Lactose treatment, which competes for galectin binding, or Gal3 knockdown abolished this increase, and addition of 1 or 10 μg/mL exogenous Gal3 increased MHCI internalization [62]
CD59	*Global* Supplementation with GlcNAc (increased glycan branching)	Constitutive	30 min	Acid wash	Decreased internalization in HeLa cells upon supplementation with GlcNAc [62]. On the contrary, increased internalization upon supplementation with GlcNAc in Beas2b cells [62]	Addition of 1 or 10 μg/mL exogenous Gal3 led to a decrease in CD59 antibody internalization in HeLa. Gal3 depletion increased CD59 internalization. Inhibition of all galectin–glycans interactions through lactose treatment however decreased CD59 internalization [62]
Neuropilin-1	*Specific* Splicing variants lacking the N-glycosylation sites N150 or N261	Constitutive	15 min	Reversible cell surface biotinylation	Increased internalization of splicing variants compared to full-length protein [154]	Increased perinuclear localization of mutants N150Q, N261Q, and N150-261Q compared to WT. These mutants furthermore colocalized with endosomal markers [154]
CCR7	*Specific* Mutants N36A, N292A, and N36-292A	Ligand-induced (0.5 μg/mL CCL19)	30 min	Differential staining of cell surface and intracellular CCR7	Decreased internalization of N36A and N36-292A, but not N292A compared to WT [156]	
Neurokinin receptor	*Specific* Mutants N14Q, N18Q, and N14-18Q	Ligand-induced (62 pM ^125^I-BH-SP)	5 min	Acid wash	Increased internalization of N14-18Q, compared to single mutants and WT receptor [158]	
